# IMRT and HDR-ICBT for Locally Advanced Clear Cell Adenocarcinoma of the Cervix in Uterus Didelphys Associated With Unilateral Renal Agenesis

**DOI:** 10.3389/fonc.2020.01136

**Published:** 2020-07-29

**Authors:** Chengzhi Lei, Manni Huang, Ning Li, Jusheng An, Suiyang Xiong, Xiaoguang Li, Lingying Wu

**Affiliations:** Department of Gynecologic Oncology, National Cancer Center/National Clinical Research Center for Cancer/Cancer Hospital, Chinese Academy of Medical Science and Peking Union Medical College, Beijing, China

**Keywords:** clear cell adenocarcinoma, genitourinary malformation, uterus didelphys, unilateral renal agenesis, cervical adenocarcinoma

## Abstract

Clear cell adenocarcinoma of the cervix (CCAC) with genitourinary malformations is rare. Here, we report a case of CCAC in uterus didelphys (UD) associated with unilateral renal agenesis (URA) that was treated with intensity-modulated radiotherapy (IMRT) and high-dose rate intracavitary brachytherapy (HDR-ICBT). We also retrospectively reviewed the medical records of CCAC cases with genitourinary malformations treated at the National Cancer Center/Cancer Hospital (Beijing, China) between December 2006 and June 2017. Eight cases of this rare condition were identified by pathologic diagnosis. Seven patients received surgical treatment including radical hysterectomy (*n* = 4), modified radical hysterectomy (*n* = 1), and total hysterectomy (*n* = 2). Five patients received adjuvant radiotherapy and chemotherapy after surgery. One patient with CCAC in UD associated with URA was treated with radical IMRT and adjuvant chemotherapy. The eight patients were followed up for an average of 7.9 years; in seven cases, there was no evidence of disease recurrence, while one patient relapsed and died after 1.5 years of treatment. On the basis of these findings, locally advanced CCAC in UD associated with URA can be effectively treated with radical IMRT.

## Introduction

Clear cell adenocarcinoma of the cervix (CCAC) is rare, accounting for 4–9% of cervical adenocarcinomas (1). CCAC has the same histology as clear cell adenocarcinomas (CCAs) of the endometrium, ovary, and vagina, which are associated with Müllerian duct abnormalities (2). Congenital Müllerian anomaly (CMA) is diagnosed in 2–4% of women with a normal reproductive outcome and is frequently associated with uterus didelphys (UD) (3). CCAC in UD associated with unilateral renal agenesis (URA) is extremely rare.

Here, we report a case of CCAC in UD associated with URA treated with radical intensity-modulated radiotherapy (IMRT) and high-dose rate intracavitary brachytherapy (HDR-ICBT) at the National Cancer Center/Cancer Hospital (Beijing, China) between December 2016 and June 2017. The patient's medical records were retrospectively reviewed, and we also carried out a comprehensive review of seven cases with this rare joint condition reported in the literature (4–8). Clinicopathologic features, treatments, and outcomes for the eight cases are shown in Table 1.

**Table 1:** Clinical features of eight patients with CCAC and CMA reported in the literature and treated at the NCC/CH.

**No**.	**Age (years)**	**Gravida/para**	**DES**	**Site/stage**	**Path**	**Genitourinary malformation**	**Treatment**	**Follow-up**		**First author, year**
								**Time (years)**	**Outcome**	
1	34	NA	N	Cervix/IIA	CCAC	UD double vagina URA	RH + PV + PLA	24	NED	Nordqvist, 1976
2	27	NA	N	Cervix/IB	CCAC	UD double vagina URA	RH Intracavitary + pelvic radiation Local recurrence after 3 months PV + PLA Uretrovaginal fistula, nephrostomy	16	NED	Nordqvist, 1976
3	49	G2P2	N	Cervix/IIA	CCAC	UD 2 cervix URA	RH + BSO + PLA EBPR: 56 Gy + vaginal irradiation: 16 Gy	4.5	NED	Spörri, 2000
4	33	G1P1		Cervix/IB1	CCAC	UD 2 cervix URA	MRH + RSO + PLA EBPR: 50 Gy + chemotherapy (PAC ×6)	10	NED	Kawano, 2013
5	65	G3P2	N	Cervix/?	CCAC	UD 2 cervix URA	TLH + BSO + PLA Concurrent chemoradiation therapy	1.0	NED	Kusunoki, 2018
6	20	NA	NA	Vagina right cervix/I	CCAC	HWWS (URA)	LRH + BSO + PLA + TV	3.0	NED	Zong, 2019
7	31	NA	NA	Double cervix Vaginal septum/IIA	CCAC	HWWS (URA)	Chemotherapy (TP × 3), LH + BSO + PPLA, chemotherapy (TP × 2), CCRT Local recurrence and distant metastases after 4 months; chemotherapy (TC × 3) and PE	1.5	DOD	Zong, 2019
8	65	G4P3	N	Cervix/IIB	CCAC	UD 2 cervix URA	Chemotherapy (TP × 3), RT	3.2	NED	Present case

*BSO, bilateral salpingo-oophorectomy; CCAC, clear cell adenocarcinoma; CCRT, concomitant chemoradiotherapy; CMA, congenital Müllerian anomaly; DES, diethylstilbestrol; EBPR, external-beam pelvic radiation; HWWS, Herlyn–Werner–Wunderlich syndrome; MRH, modified radical hysterectomy; N, no; NA, not available; NCC/CH, National Cancer Center/Cancer Hospital; NED, no evidence of disease; PAC, cisplatin plus adriamycin plus cyclophosphamide; PE, pelvic exenteration; PLA, pelvic lymphadenectomy; PPLA, pelvic-paraaortic lymphadenectomy; PV, partial vaginectomy; RH, radical hysterectomy; RSO, right salpingo-oophorectomy; RT, radical radiotherapy; TC, paclitaxel plus carboplatin; TLH, total laparoscopic hysterectomy; TP, pacitaxel plus platinum; UD, uterus didelphys; URA, unilateral renal agenesis*.

## Case History

The patient was a 65-year-old female, gravida 4/para 3 with no abnormalities during her deliveries. The limited record of her medical history showed no *in utero* diethylstilbestrol (DES) exposure. Left modified radical mastectomy was performed on April 29, 2014. Postoperative histopathology confirmed infiltrating ductal carcinoma of the breast. The clinical stage (pTNM) was T2N1M0. There was strong positive staining for HER2, but negative staining for ER and PR by immunohistochemistry.

Following the surgery, the patient was treated with a docetaxel/carboplatin/trastuzumab regimen for six cycles and achieved complete clinical remission after completion of therapy. No tamoxifen or aromatase treatment was used.

The patient was transferred to our institution on October 12, 2016 (15 years after menopause), after experiencing genital bleeding for 1 month. Diagnostic curettage was performed along with histopathologic analysis of an endometrial polyp. Speculum examination revealed mild ulceration of the cervix (~2 cm), and pelvic and rectal examinations showed right parametrial extension, with the patient complaining of pain during pelvic examination. A single cervix was observed by colposcopy under anesthesia. Pathologic analysis of a cervical biopsy suggested CCA. There was strong positive staining for cervical markers (e.g., PAX8 and P53) by immunohistochemistry. A computed tomography (CT) scan of the chest and abdomen revealed UD, left renal agenesis, and no evidence of lymph node metastasis or distant metastasis. Magnetic resonance imaging (MRI) showed bilateral uterine malformation and local fusion of bilateral cervix. Abnormal nodules 2.5 cm in diameter were observed in the right parametrial area, along with polyps 1.5 cm in diameter in the uterine cavity. The human papillomavirus (HPV) test was negative. Tumor makers such as SCC, CA125, and CA19-9 were within normal limits. The diagnosis was CCAC IIB (Fédération Internationale de Gynécologie et d'Obstétrique classification).

## Radiotherapy and Chemotherapy

**Figure 1:**
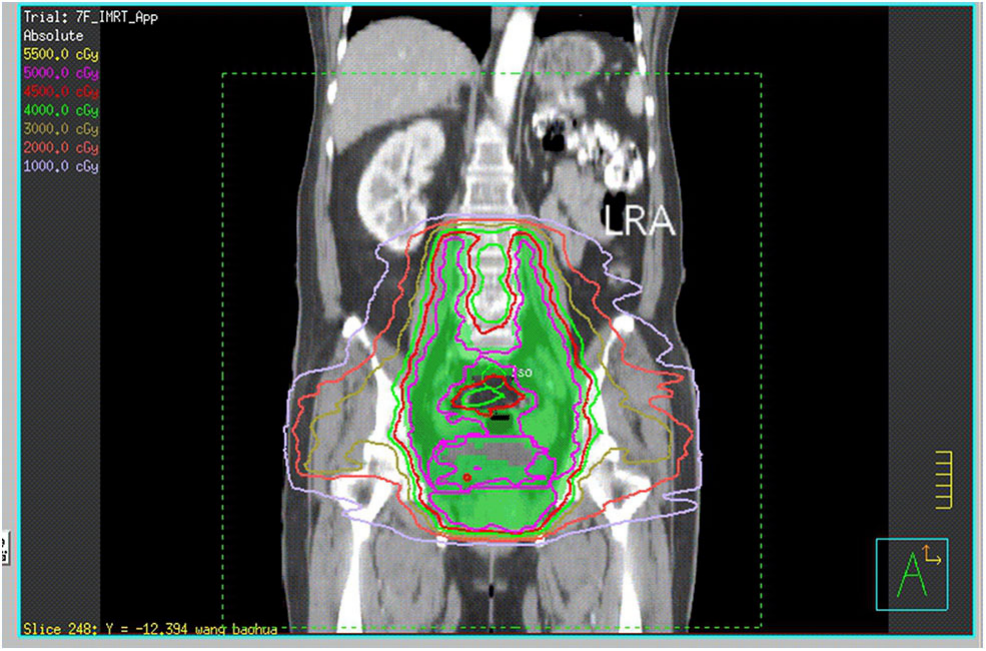
CT reconstruction of the coronal plane shows the upper and caudal field borders. LRA, left renal agenesis.

**Figure 2:**
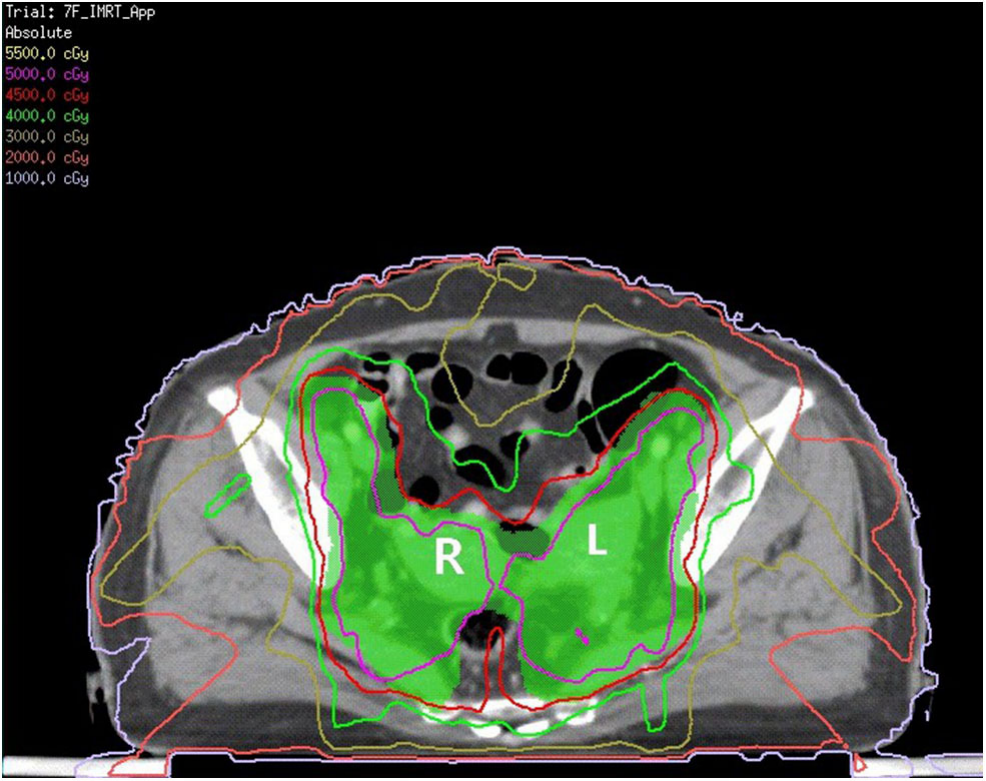
The green area is shown as PTV. The red line is the 4,500 cGy isodose curve. L, left uterine; R, right uterine.

The patient received neoadjuvant chemotherapy [two cycles of paclitaxel (175 mg/m^2^) and carboplatin (area under the receiver operating characteristic curve four)] from December 9, 2016, to January 12, 2017, followed by whole-pelvic CT-based IMRT. The clinical target volume (CTV) comprised the cervix, parametrium, uterus, upper third of the vagina, and regional lymph nodes (internal, external iliac, and common). The upper field border was at the level of the L4/L5 interspace, and the caudal field border was at the lower margin of the obturator foramen (Figure 1). Accounting for organ motion and setup uncertainty, we applied a 5-mm margin around the CTV to establish the planning target volume (PTV). IMRT planning consisted of three to seven coplanar fields with 6-mV photon beams. The prescription dose to cover 95% of the PTV was 45 Gy in 25 fractions (Figure 2). The following organs at risk were delineated: the spinal cord, femoral heads, kidneys, bladder, rectum, small bowel, and pelvic bone marrow.

The patient underwent definitive IMRT under anesthesia consisting of Ir-192 HDR-ICBT insertions, with a total dose of 28 Gy (four fractions at 7 Gy/week) delivered to point A. The doses were referred to an adapted point A, 2 cm lateral and superior to each cervical structure. ICBT was performed by placing a tandem 6 cm in length into one side of the uterine canal, with two fractions of 7 Gy delivered to both the right and left sides. Complete response was achieved with therapy.

## Follow-up

After nearly 3.2 years of follow-up, there were no clinical or radiologic signs (by MRI performed every 6 months) of local or locoregional relapse. Acute toxicity was mild and there has been no significant late toxicity since the completion of treatment, as evaluated according to the National Cancer Institute Common Terminology Criteria for Adverse Events (CTCAE) v4.03.

## Discussion

UD, which has a reported incidence of 1:1,026 (9), is caused by the failure of Müllerian duct fusion during development and may be accompanied by genitourinary malformation. UD frequently co-occurs with URA, with an incidence of 31.8%. However, CCAC associated with both UD and URA is extremely rare.

Although the etiology of CCAC is unknown, the currently held view is that a history of intrauterine exposure to DES plays a role based on a report that intrauterine exposure to non-steroidal estrogens, particularly DES, predisposed young women to the development of CCA (10). The average age of non-DES-related CCAC is 50 years; the peak incidence of DES-related CCAC varies across age groups, with the first peak occurring between 17 and 37 years old and the second between 44 and 88 years old (11). Of the eight patients in our case review, five and three, respectively, were in these age ranges, and the mean age was 40.5 years old. As an estrogen, DES interferes with the normal process of differentiation and degeneration of the Müllerian epithelium in the fetal vagina. The persistence of Müllerian cells altered at the subcellular level can potentially lead to the development of carcinoma in later life (6). However, a similar sequence of events must also occur spontaneously, as CCA can develop in women without a history of maternal estrogen exposure (11). Use of DES during pregnancy has been banned for the last 40 years, and the correlation between CCAC incidence and history of DES exposure is now clinically insignificant. In our review of eight cases, five had no clear history of DES exposure, and no information was available in three. DES is not a potent carcinogen, and other factors contribute to the pathogenesis of CCA of the vagina and cervix (12) such as genetics (e.g., p53 gene mutation), instability of microsatellite repeat sequences, HPV infection, Bcl-2 protein overexpression, and exogenous factors (13–16). The patient treated at our hospital was PAX8(+ + +) and P53(++) by immunohistochemistry and tested negative for HPV.

The clear cells of the cervix are thought to arise from mesonephric and Müllerian ducts (7). Some researchers have proposed that congenital malformations of the genitourinary system are related to the occurrence of CCAC. All eight patients reviewed in the present report had unilateral renal deficiency in addition to UD, suggesting that patients with simultaneous malformations of the genital and urinary tracts have a high risk of developing CCAC. Patients with congenital malformations of the genitourinary system are prone to multiple primary malignancies; a case of simultaneous uterine and renal cell carcinoma in an elderly woman with a septate vagina, double cervix, UD, and a single kidney secondary to contralateral renal agenesis has been reported (17). In our study, one patient with URA and bilateral uterus had CCA of the kidney and mucinous carcinoma of the breast, while another with UD and URA had breast cancer (8). However, because of the rarity of these cases and lack of genetic information, conclusions cannot be drawn regarding the development of carcinoma in patients with congenital genitourinary anomalies.

The main clinical symptom of CCAC is irregular vaginal bleeding accompanied by abdominal discomfort (18). Pelvic examination is difficult and painful for patients with genital tract malformations. Because occult lesions may be associated with UD, early detection of cervical abnormalities is challenging; therefore, gynecologic examinations can be performed under anesthesia along with pelvic MRI for patients with CMA. Given the low incidence of CCAC combined with CMA, there is no standardized treatment, which often targets the CCAC. At early stages, radical hysterectomy and pelvic lymph node resection are appropriate; radical trachelectomy may be considered for patients who wish to preserve their fertility (19). However, in CCAC combined with CMA (especially URA), anatomic abnormalities increase the risks associated with surgery. The ureter on the healthy side should be strictly preserved during the operation. In our review, there were three cases of stage I, four of stage II, and one with undetermined stage. Four patients underwent radical hysterectomy, one underwent modified radical hysterectomy, and two underwent total hysterectomy.

In patients with adverse prognostic factors, surgery is followed by chemotherapy and/or radiotherapy. For locally advanced stage II CCAC, neoadjuvant chemotherapy can improve the success rate of surgical resection and enhance sensitivity to radiotherapy (20). One patient in our case review received radical radiotherapy, and five received pelvic radiotherapy after surgery. Chemotherapy and/or radiotherapy are also treatment options for advanced-stage and inoperable patients. However, there have been few studies on the efficacy of radiotherapy or chemotherapy for CCAC, and the findings are controversial. A Japanese study reported a 5-year overall survival rate of 20.2% in patients with stage IIIB CCAC treated by HDR-ICBT combined with external beam radiation therapy, which is far lower than the rate in patients with cervical squamous cell carcinoma (47.2–55.2%) (20). CCAC combined with CMA is very difficult to treat with ICBT because of the anatomic abnormalities and pain caused by applicator placement. It is therefore recommended that ICBT be performed under anesthesia. As there are no commercialized applicators available for UD, if ICBT is performed by placing a tandem into one side of the uterine canal, an insufficient radiation dose will be delivered to point A (2 cm lateral and superior to each cervical structure). In our patient, we used the mold technique in the absence of an applicator; after insertion of a tandem 6 cm in length into one side of the uterine canal, four fractions of 7 Gy were delivered (two fractions to each of the right and left sides). After nearly 3.2 years of follow-up, there was no local recurrence. Given the rarity of CCAC with CMA, there is little information on prognosis (21). The eight patients in our review were followed up for 1–24 years, with an average follow-up time of 7.9 years; in seven cases, there was no evidence of disease recurrence, while one patient relapsed and died after 1.5 years of treatment.

In conclusion, the treatment of CCAC with genitourinary malformations is clinically challenging because of the rarity of this condition. IMRT and HDR-ICBT can be used to treat cases of locally advanced CCAC in UD associated with URA.

## Data Availability Statement

The raw data supporting the conclusions of this article will be made available by the authors, without undue reservation.

## Ethics Statement

The studies involving human participants were reviewed and approved by the Cancer Hospital, Chinese Academy of Medical Sciences. The patients/participants provided their written informed consent to participate in this study. Written informed consent was obtained from the individual(s) for the publication of any potentially identifiable images or data included in this article.

## Author Contributions

CL and MH conceived the study and wrote the manuscript. NL, JA, XL, and SX collected and analyzed the data. LW provided expert clinical knowledge. All authors critically edited the manuscript for intellectual content.

## Conflict of Interest

The authors declare that the research was conducted in the absence of any commercial or financial relationships that could be construed as a potential conflict of interest.
